# Non bulimic shitty meal

**DOI:** 10.1192/j.eurpsy.2021.1854

**Published:** 2021-08-13

**Authors:** A. Costa, S. Jesus, J. Alcafache

**Affiliations:** Psiquiatria E Saúde Mental, Centro Hospitalar do Baixo Vouga, Aveiro, Portugal

**Keywords:** Coprophagia, Feces, cognitive impairment, schizophrénia

## Abstract

**Introduction:**

Coprophagia is a relatively rare phenomenon characterized by the ingestion of feces, and it is usually classified as a rare form of pica. It has been associated with multiple organic causes or mental disorders such as brain tumors, alcoholism, mental retardation, dementia, schizophrenia, depressive disorders or fetishism.

**Objectives:**

Case report and reflection on its etiology

**Methods:**

A Pubmed search was performed with the MeSH terms “Coprophagy” and “pica”. Relevant articles obtained from the respective bibliographic references were also consulted.

**Results:**

A 56-year-old man with a history of psychiatric follow-up with a diagnosis of schizophrenia and cognitive impairment, assessed for behavioral changes such as cat feces intake. After possible organic causes were excluded, treatment with supportive psychotherapy and pharmacologically began with a selective serotonin reuptake inhibitor, fluoxetine, along with treatment for schizophrenia.

**Conclusions:**

According to literature, coprophagia often occurs associated with other medical or neuropsychiatric conditions. Although the etiology, pathophysiology and management remains unclear, several pharmacologic treatments have been attempted with some degree of success. We describe a case of unusual behavior, coprophagia, associated with cognitive impairment and schizophrenia that responded favorably to fluoxetine although without complete remission, in order to contribute to a future nosological redefinition.

**Disclosure:**

No significant relationships.

